# Segmentation of Cerebrovascular Anatomy from TOF-MRA Using Length-Strained Enhancement and Random Walker

**DOI:** 10.1155/2020/9347215

**Published:** 2020-09-19

**Authors:** Ruoxiu Xiao, Cheng Chen, Hanying Zou, Ying Luo, Jiayu Wang, Muxi Zha, Ming-An Yu

**Affiliations:** ^1^School of Computer and Communication Engineering, University of Science and Technology Beijing, Beijing 100083, China; ^2^Institute of Artificial Intelligence, University of Science and Technology Beijing, Beijing 100083, China; ^3^School of Energy and Environmental Engineering, University of Science and Technology Beijing, Beijing 100083, China; ^4^Department of Interventional Ultrasound, China-Japan Friendship Hospital, Beijing 100029, China

## Abstract

Cerebrovascular rupture can cause a severe stroke. Three-dimensional time-of-flight (TOF) magnetic resonance angiography (MRA) is a common method of obtaining vascular information. This work proposes a fully automated segmentation method for extracting the vascular anatomy from TOF-MRA. The steps of the method are as follows. First, the brain is extracted on the basis of regional growth and path planning. Next, the brain's highlighted connected area is explored to obtain seed point information, and the Hessian matrix is used to enhance the contrast of image. Finally, a random walker combined with seed points and enhanced images is used to complete vascular anatomy segmentation. The method is tested using 12 sets of data and compared with two traditional vascular segmentation methods. Results show that the described method obtains an average Dice coefficient of 90.68%, and better results were obtained in comparison with the traditional methods.

## 1. Introduction

Vascular malformations caused by vascular stenosis and aneurysms have become the leading cause of cerebrovascular diseases [1] and pose a significant threat to human health. Time-of-flight (TOF) magnetic resonance angiography (MRA) is a clinical cerebrovascular angiography technology with noninvasive, rapid, and high-resolution characteristics and has been widely used in the diagnosis and treatment of cerebrovascular diseases. When multiple scales of blood vessels, image noise, and uneven contrast are present, obtaining anatomical structures of precise blood vessels from TOF images is critical for the diagnosis and quantitative analysis of cerebrovascular diseases. Moreover, accurate cerebrovascular segmentation is an essential prerequisite for neurosurgical planning and navigation. Therefore, designing an accurate segmentation of cerebrovascular vessels has received extensive attention from researchers in related fields.

This work proposes an automatic algorithm to obtain seed points in the TOF-MRA image and overcome the difficulties of the abovementioned methods. Given that the blood vessel branching volume is usually small and the contrast is low, blood vessels are difficult to detect in the original TOF image; thus, the Hessian matrix of the TOF-MRA image of the multiscale space is used to calculate the enhanced blood vessel image. At last, the vascular structure is segmented on the enhanced blood vessel image via the random walker method in combination with the acquired seed points. The main contributions are as follows: First, a fully automatic cerebrovascular segmentation method is proposed, and a control experiment is designed to verify the segmentation accuracy. Second, the proposed length-strained enhancement method can effectively improve the segmentation accuracy. Finally, the influence of the random walker parameter was explored, and the best plan was applied.

This work is organized as follows. Related research work is explored in Section 2. A detailed description of the proposed method is presented in Section 3. Experiments and the overall performance are introduced in Section 4. Finally, Section 5 discusses the conclusion.

## 2. Related Work

Many methods for segmenting 3D cerebrovascular structures from TOF-MRA images have been proposed [2]. Common methods include tracking-based segmentation, statistical model-based methods, and neural network-based methods.

Tracking-based methods typically track adjacent edges sequentially from a point in the image through a specific search mechanism. All search processes are completed by following a given termination condition to ultimately capture the entire vascular structure. This approach often achieves good results when combined with connectivity information and edge detection techniques. Schneider et al. [3] proposed a new joint 3D vessel segmentation and centerline extraction framework based on multivariate Hough voting and tilted random forest (RF) by learning from noisy annotations. This method relies on steerable filters to efficiently compute the local image features of different scales and orientations. Wei et al. [4] introduced a grid centerline extraction method that combines a series of advanced techniques in branch segmentation schemes and discrete geometry processing to solve the challenging problem of vascular centerline extraction. Oliveira et al. [5] iteratively tracked a whole vascular network by using a single starting point in the basis of a sample point cloud distributed over a concentric spherical layer. A container model and a sample point matching degree with the model were proposed. Network tracking is implemented as a minimum cost flow problem, and a novel optimization scheme is proposed to iteratively track vascular structures by inherently processing the bifurcation and path. Because the tracking-based vascular structure segmentation algorithm is based on the blood vessels with continuous structural features, the high integrity of the vascular structure is required. The method must also specify the initial point as the starting point of the local operator and is highly dependent on the initial parameters.

The statistical model-based approach is a typical method of cerebrovascular segmentation based on the principles of Bayesian statistical classification. This method constructs two gray distribution functions to fit the background and blood vessels in the image. The threshold of vessel segmentation is obtained by optimizing the grayscale distribution function. Wen et al. [6] proposed a method based on automatic statistical strength to extract 3D cerebrovascular structures from TOF-MRA data. The intensity histogram of the brain image sequence is fitted using a finite mixed model in which the cerebrovascular structure is modeled by a Gaussian distribution function, while the Gaussian and Rayleigh distribution functions model other low-intensity tissues. Lu et al. [7] used a multiscale filtering algorithm to enhance the blood vessels and suppress noise, thereby enabling new statistical features for filtered data. A hybrid model formed by three probability distributions (two exponential distributions and one Gaussian distribution) is established to fit a histogram curve of the filtered data, wherein an expectation maximization (EM) algorithm is used for parameter estimation. Finally, a 3D Markov random field is used to improve the accuracy of pixel classification and posterior probability estimation. Lu et al. [8] proposed an improved variation level set method that uses nonlocal robust statistics to suppress the effects of noise in MR images. Nonlocal robust statistics representing vascular features are learned adaptively from the seeds provided by the user, and *K*-means clustering in the seed neighborhood is used to exclude seeds that are affected by noise. The neighborhood of the appropriate seed is placed in the array to calculate nonlocal robust statistics, and a variation level set can be constructed. Deviation correction is used in the level set formulation to reduce the effect of intensity nonuniformity of the MRI. Given that the blood vessels are mainly located in the high-intensity region of the TOF-MRA data set, the high-intensity large blood vessels can be easily distinguished when a blood vessel is segmented using a statistical model-based method. However, given their low intensity, the small blood vessels are difficult to identify by using statistical models.

A deep convolutional neural network is a common method in processing cerebrovascular segmentation in TOF-MRA images. Image segmentation and vascular extraction methods based on neural networks are mainly used to simulate the learning process of biology, and many elements simulating the mechanism of biological learning constitute a network. Driven by the blood vessel calibration data set, the network parameters are gradually converged to obtain the final network structure for use in the segmentation of the blood vessels. Liskowski and Krawiec [9] proposed a supervised segmentation technique using deep neural networks to train large (up to 400,000) samples. These samples are normalized by global contrast, zero-phase whitened, and enhanced with geometric transformations and gamma correction. Several variations of this approach, including structured prediction, where the network simultaneously classifies multiple pixels, have been considered. Dasgupta and Singh [10] developed the segmentation task as a multitag reasoning task and exploited the implicit advantages of convolutional neural networks (CNN) combined with structured prediction. Fu et al. [11] developed the vascular segmentation problem as a boundary detection task and solved it by using a novel deep learning architecture. The approach is based on two key ideas: the application of multiscale and multilevel CNN with side output layers to learn rich hierarchical representations and the remote interaction between conditional random field (CRF) analog pixels. CNN and CRF layers are combined into an integrated deep network called DeepVessel. The neural network method relies on the data set to obtain convergence, which requires a large amount of labeled data. In practical applications, this technique depends on the operating speed and storage capacity of the device. However, given that numerous studies are mainly based on slide by slide, obtaining all 3D texture information effectively is impossible. Therefore, accurate and low-cost segmentation is a significant problem in neural network methods.

## 3. Methods

**Figure 1:**
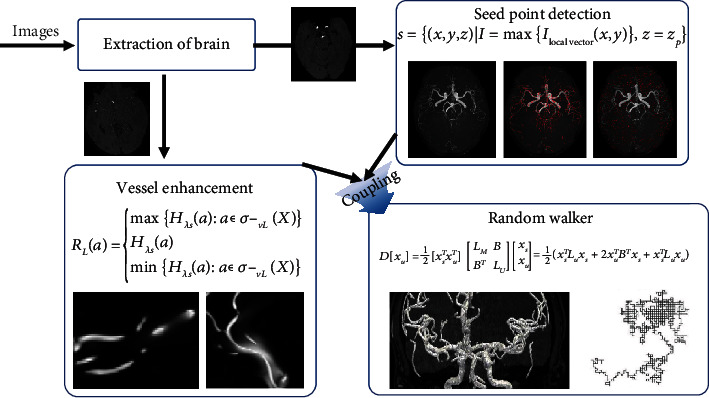
Algorithm flowchart.

As can been seen from Figure 1, the main steps of the proposed automatic blood vessel segmentation method can be divided into the following four parts. First, the brain features in the TOF-MRA image are combined, and the brain region is extracted on the basis of the region growth and the cost-optimal path. Second, a set of seed points is automatically obtained in conjunction with the high-brightness structure and connectivity of the blood vessels. Third, spatial multiscale angiographic enhancement maps are constructed and special judgments are made on the basis of noise and small structures. Finally, the segmented 3D vascular structure is obtained via the random walker method based on the detected seed point set and vascular enhanced image.

### 3.1. Extraction of the Brain

The region of interest in the brain-filled region should be located to obtain an accurate cerebrovascular image. The present work uses regional growth to initially segment low-intensity tissues adjacent to the brain, such as the skull and cerebrospinal fluid regions. Thus, the threshold ranges and seed points for seed growth must be defined. The gray histogram of the TOF slice is calculated and divided into two regions, namely, the brain and the nonbrain regions. A mixed Gaussian model is established to simulate the distribution of the histogram, where *i* = 1, 2, ⋯, *m* indicating the number of samples. The gray value is the only feature. The *K* value is 2, which corresponds to two regions: one part represents the skull (the background of which is of low intensity), and the other part corresponds to the blood vessels, brain, nose, and eye area. A previous work [12] estimated the parameters by using the expected maximum method. However, in the mixed Gaussian model, the maximum likelihood function contains a logarithm and cannot be maximized via summation. In the present work, the *K* clustering method is chosen to determine the initial value. The classic Euclidean distance dist_ed_(*x*^(*i*)^, *x*^(*j*)^) is selected as a measure of distance and expressed as follows:

(1) $$\begin{array}{c} {\text{dist}}_{\text{ed}}\left(x^{\left(i\right)},x^{\left(j\right)}\right)={\left\|x^{\left(i\right)}-\left.x^{\left(j\right)}\right\|\right.}_{2}. \end{array}$$

The brain area is determined by dividing the skull and cerebrospinal fluid areas connected to it. Therefore, a good threshold range is required to achieve growth in this part of the region. A single threshold for each slice can be extracted from the hybrid model on the basis of a minimum error classification. The extracted threshold is used as the upper threshold *T*_U_ for region growth and typically contains all or most of the bone. The lower threshold *T*_L_ is defined by the lowest intensity value that appears in the middle image, which typically appears in the background area. Thus, the seed growth range *T* ∈ [*T*_L_, *T*_U_] of the region growth is obtained. The final analysis shows that the inflection point represents the skull and nose area. Therefore, the inflection point can be selected as the seed point of the region growth to complete the initial segmentation.

Given that the range of thresholds does not accurately cover all ranges, the initial segmentation has a broken structure that does not entirely enclose the brain. In accordance with a previous work [12], the support points are extracted on the basis of initial segmentation and connected using a graph-based approach. Given that the 3D surface drawing requires pillar points, 2D support points are extracted layer by layer and a path to form a closed curve is built. The center of gravity of the segmented skull is first calculated to extract these support points. From this coordinate, the rays are drawn at intervals of 22.5 degrees. The theoretical connection portion of each ray with the region is determined, and the shortest distance from the center of gravity is taken as the support point. Finally, up to 16 support points *P*_*i*_ are extracted. If no intersecting area of a ray is noted, the support points obtained by the ray are no longer considered.

The best path to the defined target node *P*_*i*+1_ is searched starting from the starting node *P*_*i*_. A cost map is constructed from the slice image to connect the extracted support points. Here, the pixels of the image are represented as graphic nodes, and edges are created between each pixel and its eight neighbors. The cost-optimal path is then defined as the path with the least cost, which consists of the sum of the cost of each edge accessed from the path to the target node. The included features are as follows: Laplacian zero-crossing *f*_*z*_, gradient magnitude*f*_*G*_, and gradient direction*f*_*D*_ [13]. These cost terms are weighted together to form the cost of the edge *l*(*p*, *q*) between nodes *p* and *q* as follows:

(2) $$\begin{array}{c} l\left(p,q\right)=\varpi_{G}\cdot f_{G}\left(q\right)+\varpi_{Z}\cdot f_{Z}\left(q\right)+\varpi_{D}\cdot f_{D}\left(q\right). \end{array}$$

**Figure 2:**
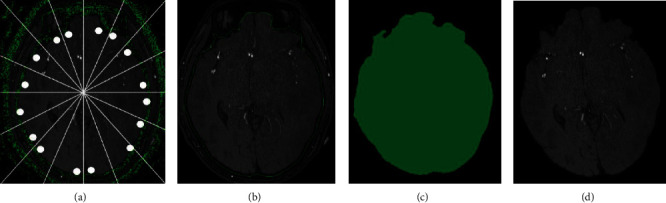
Brain extraction: (a) detection of support points; (b) generation of brain boundary by support points; (c) obtained brain mask; (d) extracted brain structure.

The presence of bone marrow interference leads to incorrect segmentation. Considering that the change between the adjacent layers is negligible, the shortest distance between the pixel points of each layer and the adjacent layer is calculated, and the absolute value therein is taken as the distance *d* between adjacent layers. The dividing line is taken as 50 subregion *D* in succession. When the minimum distance of the subarea still satisfies *d*′ > *d*, *D* is considered as the disturbed area. The cost of *D* is recalculated, expanding from field 9 to field 25, and a new path is regained.  Figure 2 shows all the steps above.

### 3.2. Seed Point Detection

Seed points are often needed to be selected as the end of walk of the random walker [14]. A sufficient number of seed points in the target range should be ensured to increase the accuracy of the probability calculation. The nonconnected area caused by the abnormal situation, such as the lesion area and image quality interference, should be marked separately similar to a previous study [15]; the prior probability is used to improve the segmentation accuracy of the fracture area. Given that most of the blood vessels in the image are characterized by small structures and blurred textures, traditional manual labeling is time consuming and prone to omission or mislabeling.

Inspired by [16], an automatic selection scheme for seed points is designed in the present study. The method combines the following prior knowledge: (1) The angiographic structure of the contrast-enhanced blood vessel in the image exhibits a high-gradation gray value and has a significant boundary gradient characteristic; thus, the blood vessel portion can be obtained accurately under a sufficient threshold constraint. (2) In the 3D view, voxel labeling is easily subjected to differences in image depth and the parallax error is judged. The traditional labeling usually adopts the layer-by-layer processing method until the labeling ends. (3) In the image, the blood vessels are usually presented in a tubular structure of a connected region.

Based on the above principles, the projection of maximum intensity preserves the highlighted vascular area. We project from angle *X* to *Y* to *Z* to avoid occlusion between the blood vessels.

**Figure 3:**
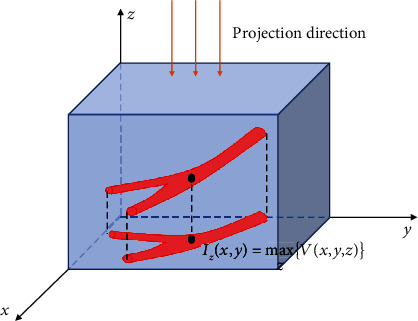
Schematic of the maximum intensity projection method.

As can been seen in Figure 3, 2D projection map *I*_*z*_(*x*, *y*) on the *XY* plane can be obtained in the *Z*-axis direction. The pixel value of each point is determined by the maximum gray value in the *Z*-axis direction. The *Z* coordinate of each projection point is saved as the hidden variable *Z*_*p*_ of the point to satisfy $Z_{p}\left(x,y\right)=\underset{z}{\arg\max}\left\{V\left(x,y,z\right)\right\}$.

(3) $$\begin{array}{c} I_{z}\left(x,y\right)=\underset{z}{\max}\left\{V\left(x,y,z\right)\right\}. \end{array}$$

A highlighted blood vessel partial region *U*_ini_ is obtained in *I*_*z*_(*x*, *y*) on the basis of the gray limit. The connected domain *V*, which is defined as the connected domain part, should be detected to avoid the interference caused by local noise points. Thus, the connected domain set *V* = {*V*_1_, *V*_2_, ⋯, *V*_*N*_} in *U*_ini_ can be obtained. Noise is usually only present in the interlayer image; thus, *V* can determine the parts that are mainly blood vessels. Finally, the local maximum is extracted as the seed point to further screen and eliminate the interference, thereby satisfying the following condition:

(4) $$\begin{array}{c} S=\left\{\left(x,y,z\right)\;|\;I=\max\left\{I_{\text{local vector}}\left(x,y\right)\right\},z=z_{p}\right\}, \end{array}$$

where $I_{\text{local vector}}=\underset{\Delta x\;=\;-1,0,1\text{ and }\Delta y\;=\;-1,0,1}{I_{z}\left(x+\Delta x,y+\Delta x\right)}$ is the image area of size.

If some blood vessels overlap in the MIP direction in the projected image, their blood vessel information and number of detected seed points will decrease. The problem of vascular information in the same direction is overcome in the present work by simultaneously projecting the *X*- and *Y*-axes and obtaining the seed point sets *U*_*sx*_ and *U*_*sy*_ from the *YZ* and *XZ* projection planes, respectively. Finally, the seed point set *U*_*s*_ is obtained to satisfy *U*_*s*_ = *U*_*sx*_ + *U*_*sy*_ + *U*_*sz*_.

### 3.3. Vessel Enhancement

The image detection structure is obtained by conducting feature analysis of the Hessian matrix to capture the second-order structure of the local intensity variation near each pixel, and the Hessian matrix of the 3D medical image *I*(*x*, *y*, *z*) is constructed [17] and expressed as follows:

(5) $$\begin{array}{c} H=\nabla^{2}I=\left(\begin{array}{ccc} I_{xx} & I_{xy} & I_{xz}\\ I_{yx} & I_{yy} & I_{yz}\\ I_{zx} & I_{zy} & I_{zz} \end{array}\right), \end{array}$$

where *I*_*xx*_, *I*_*xy*_, *I*_*xz*_, ⋯, *I*_*zz*_ correspond to the second-order partial differentials of *I*(*x*, *y*, *z*), respectively.

In digital images, the second-order partial differentials in the *X*, *Y*, and *Z* directions are represented in discrete ways:

(6) $$\begin{array}{c} \left\{\begin{array}{c} I_{xx}=\frac{\partial^{2}I}{\partial x^{2}}=I\left(x-1,y,z\right)+I\left(x+1,y,z\right)-2I\left(x,y,z\right),\\ I_{yy}=\frac{\partial^{2}I}{\partial y^{2}}=I\left(x,y-1,z\right)+I\left(x,y+1,z\right)-2I\left(x,y,z\right),\\ I_{zz}=\frac{\partial^{2}I}{\partial z^{2}}=I\left(x,y,z-1\right)+I\left(x,y,z+1\right)-2I\left(x,y,z\right). \end{array}\right. \end{array}$$

The corresponding mixed partial differential can be expressed as follows:

(7) $$\begin{array}{c} \left\{\begin{array}{c} I_{xy}=I_{yx}=\frac{\partial^{2}I}{\partial x\partial y}=I\left(x+1,y+1,z\right)+I\left(x,y,z\right)-I\left(x+1,y,z\right)-I\left(x,y+1,z\right),\\ I_{yz}=I_{zy}=\frac{\partial^{2}I}{\partial y\partial z}=I\left(x,y+1,z+1\right)+I\left(x,y,z\right)-I\left(x,y+1,z\right)-I\left(x,y,z+1\right),\\ I_{xz}=I_{zx}=\frac{\partial^{2}I}{\partial x\partial z}=I\left(x+1,y,z+1\right)+I\left(x,y,z\right)-I\left(x+1,y,z\right)-I\left(x,y,z+1\right). \end{array}\right. \end{array}$$

Given that the blood vessels usually have different sizes, the eigenvalues of multiscale Hessian matrices are usually analyzed. The Hessian matrix precisely measures the contrast between the inner and outer regions (−*s*, *s*), which indicates that the scale *s* can represent the radius of the blood vessel. The Hessian matrix is a symmetric matrix, and its eigenvalues *λ*_1_, *λ*_2_, and *λ*_3_ (|*λ*_1_| ≤ |*λ*_2_| ≤ |*λ*_3_|) are obtained by calculation.

**Figure 4:**
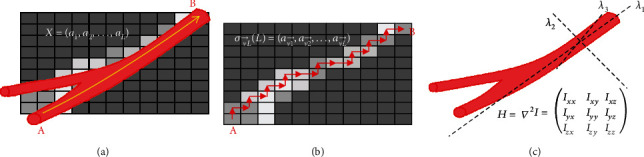
Relationship of the blood vessel and the Hessian matrix. (a) A continuous model of the blood vessel. (b) Discrete model of the blood vessel. (c) Schematic of the direction eigenvalues of the Hessian matrix at the blood vessel.

As can been seen in Figure 4, the corresponding feature vectors are $\vec{v_{1}}$, $\vec{v_{2}}$, and $\vec{v_{3}}$. *λ*_1_ represents the change in intensity along the direction of the blood vessel ($\vec{v_{1}}$), and *λ*_2_ and *λ*_3_ represent changes in intensity in the direction of the vertical vessel ($\vec{v_{2}}$ and $\vec{v_{3}}$). The blood vessels, depending on their structure, always present tall tubular structures in MRA images and are in contrast with the relatively dark background. The intensity change along the main direction of the blood vessel is considerably smaller than the intensity change along the vertical direction. A priori knowledge of this image imaging mode can be used as a consistency test to distinguish between the blood vessels and the rest of the structure. Based on this observation, the eigenvalues measure the curvature regeneration and vascular structure well. When a pixel has a large *λ*_2_ and *λ*_3_ value and a small *λ*_1_ value, it likely belongs to the blood vessel, as shown in the following equation:

(8) $$\begin{array}{c} \left\{\begin{array}{c} \left|\lambda_{1}\right|\approx 0,\\ \left|\lambda_{1}\right|\ll \left|\lambda_{2}\right|,\\ \lambda_{2}\approx \lambda_{3}<0. \end{array}\right. \end{array}$$

An adjoining sphere with a radius of 1 centered on the pixel *x*_0_ is established to quantify the differentiation criterion of the vascular structure. The Hessian matrix is mapped onto the ellipsoid structure, wherein the axial direction is given by the eigenvalue. The axial length corresponds to the eigenvalue.

Given that the ellipsoid is a second-order structure, local features can be used to reflect the image detection structure. Here, three coefficients are defined as follows: *R*_*A*_, *R*_*B*_, and *S*.

(9) $$\begin{array}{c} R_{A}=\frac{L_{a}/\pi }{{L_{s}}^{2}}=\frac{\left|\lambda_{2}\right|}{\left|\lambda_{3}\right|}, \end{array}$$

where *L*_*a*_ represents the largest cross-sectional area and *L*_*s*_ represents the length of the largest semimajor axis. The gray level invariance is maintained by its proportional relationship, and only the image geometric information is captured. This ratio can effectively distinguish between a spherical structure and a tubular structure.

(10) $$\begin{array}{c} R_{B}=\frac{V/\left(4\pi /3\right)}{L_{a}/\pi^{3/2}}=\frac{\left|\lambda_{1}\right|}{\sqrt{\left|\lambda_{2}\lambda_{3}\right|}}, \end{array}$$

where *V* represents the volume. This ratio can be used to effectively distinguish whether it is a sheet structure.

The volume occupied by the vascular structure is always small; thus, random noise may occur in the same structural features of the blood vessel. For a typical signal-to-noise ratio, the derivative of the background pixel is usually small, and the Hessian matrix norm can be written as follows:

(11) $$\begin{array}{c} S={\left\|H\right\|}_{F}=\sqrt{\overset{n}{\underset{j\leq D}{\sum }}{\lambda_{j}}^{2}}, \end{array}$$

where *D* is the dimension of the image, *H* is the Hessian matrix, and *λ*_*j*_ is the *j*th eigenvalue.

The multiscale linear filter is defined as *V*(*σ*, *x*), where *σ*_min_ and *σ*_max_ correspond to the scale, that is, the width of the blood vessel, and satisfies

(12) $$\begin{array}{c} V\left(x\right)=\underset{\sigma_{\min}<\sigma <\sigma_{\max}}{\max}V\left(\sigma ,x\right). \end{array}$$

Taylor expansion is performed on the pixel point *x* to analyze the local features of the image as follows:

(13) $$\begin{array}{c} I\left(\sigma ,x+\delta x\right)\approx I\left(\sigma ,x\right)+\delta x^{T}\nabla I\left(\sigma ,x\right)+\delta x^{T}H\left(\sigma ,x\right)\delta x. \end{array}$$

When mapping through the Hessian matrix, the eigenvalues can be decomposed and extracted into three orthogonal directions while keeping the scale factor unchanged; thus, the local second-order structure of the image is decomposable. Because our eigenvector analysis gives the direction of the minimum curvature, considering multiple directions is unnecessary when applying the filter.

Linear filters for 3D images are constructed as follows:

(14) $$\begin{array}{c} H_{\lambda s}\left(\sigma ,x\right)=\left\{\begin{array}{cc} 0, & \lambda_{2}>0\text{ or }\lambda_{3}>0,\\ \left(1-\exp\left(-\frac{{R_{A}}^{2}}{2\alpha^{2}}\right)\right)\exp\left(-\frac{{R_{B}}^{2}}{2\beta^{2}}\right)\left(1-\exp\left(-\frac{S^{2}}{2c^{2}}\right)\right), & \text{else}, \end{array}\right. \end{array}$$

where *x* is the voxel point in the volume data, *α* is the difference control parameter between the tubular structure and the disc structure, *β* is the difference control parameter of the tubular structure and the spherical structure, and *c* is the difference control parameter of the high- and low-contrast structures.

*H*_*λs*_ can enhance the effective enhancement of the vascular area but is sensitive to noise background. In order to solve this problem, nonlocal vascular path features are introduced to distinguish between the blood vessels and noise, and the details can be expressed as follows:

*(1) Adjacency and Path*. A morphological path operator was proposed in [8] for filtering curves through a specified direction. Supposing that the point set of the discrete image is *V*, the definition *a* → *b* indicates the presence of a path from point A to point B in the specified direction (Figure 4(a)). The adjacency is used to define a path of length *L*, which consists of consecutively adjacent *L* points. The point set *X* = (*a*_1_, *a*_2_, ⋯, *a*_*L*_) is referred to as a path of length *L*, and *σ*_*L*_(*X*) is used to represent it.

*(2) Vascular Path Exploration*. Inspired by the morphological path operator, we introduce directional information from the Hessian matrix to form the vascular path. Eigenvector analysis of the Hessian matrix indicates that the eigenvectors $\vec{v_{1}}$, $\vec{v_{2}}$, and $\vec{v_{3}}$ can be obtained to represent the direction along the vessel and its vertical direction. Therefore, the points in the $\vec{v_{1}}$ direction and opposite direction of each point can be merged to form a blood vessel path. Path searches for the nearest point are based on the direction of the current point and involve a point-by-point step (Figure 4(a)). The path formed by the vascular direction information of length *L* is represented by $\sigma_{\vec{v}L}$ as follows:

(15) $$\begin{array}{c} \sigma_{\vec{v}L}\left(L\right)=\left(a_{\vec{v}1},a_{\vec{v}2},\cdots ,a_{\vec{v}L}\right), \end{array}$$

where $a_{\vec{v}i}⟶a_{\vec{v}i+1}$ is the constituent element of the path. When leaving the blood vessel is possible, a stopping criterion for the path search should be established. The most obvious indicator of whether the path crosses the boundary is the local vascular response *H*_*λs*_. All vessel paths should be locally smooth, which can be enforced by limiting the change in direction between two consecutive points in the path. Thus, the condition for maintaining a vascular path search may be expressed by the following equation:

(16) $$\begin{array}{c} \left\{\left|{{\vec{v}}_{i}}^{T}{\vec{v}}_{i+1}\right|<\theta_{\text{path}}\right\}\wedge \left\{H_{\lambda s}\left(a\right)>0:a\in \sigma_{\vec{v}L}\right\}, \end{array}$$

where ${\vec{v}}_{i}$ represents the corresponding direction of the path point from the Hessian matrix. The first term in the equation forces the path to smoothen, and the second term ensures local curvature regeneration. Based on the results of vascular path analysis, the empirically chosen threshold of the smoothing constraint is *θ*_path_ = cos(*π*/6).

*(3) Length Correction*. The radius of the blood vessel usually varies along the blood vessel, especially at the bifurcation. Therefore, the length of the vascular path may be long at the center and attenuated based on the direction $\vec{v_{1}}$ after path search. However, the length of these points can be corrected by searching for another “path” from the boundary to the center along the vertical direction $\vec{v_{2}}$ and $\vec{v_{3}}$ (Figure 4(b)). The same criteria in Equation (14) are used for unification, and a radius length constraint is added as follows:

(17) $$\begin{array}{c} \left\{\left|{{\vec{v}}_{i}}^{T}{\vec{v}}_{i+1}\right|<\theta_{\text{path}}\right\}\wedge \left\{H_{\lambda s}\left(a\right)>0:a\in \sigma_{\vec{v}L}\right\}\wedge \left\{L<2s\right\}. \end{array}$$

The third item in Equation (15) ensures that the search path passes through the center point of the blood vessel. The longest length in the path is selected as the final length of the point in the same cross section of the blood vessel.

We propose a length-limited vascular enhancement as follows:

(18) $$\begin{array}{c} R_{L}\left(a\right)=\left\{\begin{array}{c} \max\left\{H_{\lambda s}\left(a\right)\text{: }a\in \sigma_{\vec{v}L}\left(X\right)\right\},\\ H_{\lambda s}\left(a\right),\\ \min\left\{H_{\lambda s}\left(a\right)\text{: }a\in \sigma_{\vec{v}L}\left(X\right)\right\}, \end{array}\right. \end{array}$$

where *L*_max_ manually sets a constant to mean the minimum length of the blood vessel and *L*_min_ is the certain maximum length of the nonvascular object. The basic idea of *R*_*L*_ is to choose the appropriate response for all points in the same vessel path; it can increase the response of long paths and suppress the response of short path points.

### 3.4. Random Walker Segmentation

**Figure 5:**
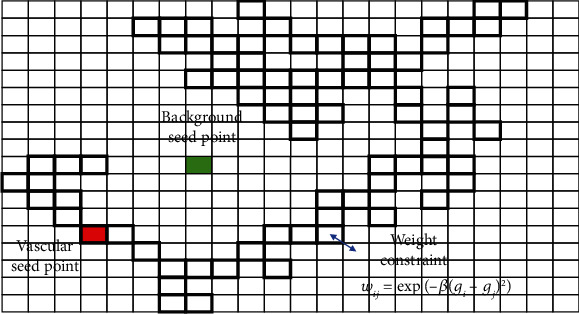
Random walk map to complete image segmentation, where the green point represents the background seed point, the red point represents the vascular seed point, and their boundary is constrained by weights.

A random walk map is constructed following the condition (Figure 5), where *V* is the set of vertices in the map, *v* ∈ *V*; *E* is the set of undirected edges of the vertices in the map, *e* ∈ *E*⊆*V* × *V*; and *e*_*ij*_ represents the connection relationship between the vertices *v*_*i*_ and *v*_*j*_. The definition of the edge weight can reflect the similarity between adjacent points, and the Gaussian weighting function [15] is selected as the edge weight as follows:

(19) $$\begin{array}{c} w_{ij}=\exp\left(-\beta {\left(g_{i}-g_{j}\right)}^{2}\right), \end{array}$$

where *g*_*i*_ is the gray value of the vertex *v*_*i*_, and *β* parameter is the influence of the gray value.

We can calculate the probability of the nonmarked point moving to the seed point which can be calculated by obtaining the edge weight, and the maximum probability is taken as the new mark of the point; finally, image segmentation can be realized. A previous work [18] proved that the process of solving probabilities can be transformed into the classical Dirichlet problem, which involves finding the harmonic function as a solution to a specified partial differential equation in a given region and taking a predetermined value on the boundary. The harmonic function satisfies Laplace's equation ∇^2^*u* = 0 and corresponds to the Euler–Lagrange equation of the Dirichlet integral *D*[*u*]; thus, the solution at which the Dirichlet integral reaches the minimum value is the desired harmonic function, where *D*[*u*] = (1/2)∫_*Ω*_|∇*u*|^2^*dΩ*.

A Laplacian matrix *L* of the map *G* is created as follows:

(20) $$\begin{array}{c} L_{ij}=\left\{\begin{array}{cc} d_{ij}, & \text{when }i=j,\\ -w_{ij}, & \text{when }v_{i}\text{ and }v_{j}\text{ are adjacent},\\ 0, & \text{others}, \end{array}\right. \end{array}$$

where *L* satisfies the condition *L* = *A*^T^*CA* and *d*_*i*_ is the degree of the vertex *v*_*i*_, that is, the sum of all the edge weights of the connected vertices,*d*_*i*_ = ∑*w*_*ij*_. *A* is an associative matrix of edges and vertices and satisfies Equation (21). *C* is the constitutive matrix of *G*, which is defined as a diagonal matrix, and the diagonal elements are the weights of the corresponding edges.

(21) $$\begin{array}{c} A_{e_{ij}v_{k}}=\left\{\begin{array}{cc} +1, & \text{when }i=k,\\ -1, & \text{when }j=k,\\ 0, & \text{others}. \end{array}\right. \end{array}$$

Therefore, a discrete form of Dirichlet's integral *D*[*u*] is obtained as follows:

(22) $$\begin{array}{c} D\left[x\right]=\frac{1}{2}{\left(Ax\right)}^{T}C\left(Ax\right)=\frac{1}{2}x^{T}Lx=\frac{1}{2}\underset{e_{ij}\in E}{\sum }w_{ij}{\left(x_{i}-x_{j}\right)}^{2}. \end{array}$$

A discrete harmonic function *x* that satisfies *D*[*x*] minimization is required. Given that *L* is a semidefinite matrix, *D*[*x*] has a unique minimum value. The vertex *V* consists of a marked point *U*_*s*_ and an unmarked point *U*_*u*_, satisfying *U*_*u*_ ∪ *U*_*s*_ = *V*, *U*_*u*_∩*U*_*s*_  =  ∅. Further decomposition of *D*[*x*] yields

(23) $$\begin{array}{c} D\left[x_{u}\right]=\frac{1}{2}\left[{x_{s}}^{T}{x_{u}}^{T}\right]\left[\begin{array}{c} L_{M}\;B\\ B^{T}\;L_{U} \end{array}\right]\left[\begin{array}{c} x_{s}\\ x_{u} \end{array}\right]=\frac{1}{2}\left({x_{s}}^{T}L_{u}x_{s}+2{x_{u}}^{T}B^{T}x_{s}+{x_{u}}^{T}L_{u}x_{u}\right), \end{array}$$

where *x*_*s*_ and *x*_*u*_ correspond to the probability of marked and unmarked points, respectively. *D*[*x*_*u*_] is solved to differentiate *x*_*u*_ and the extreme point is sought through zero: *L*_*u*_*x*_*u*_ = −*B*^*T*^*x*_*s*_. Let *x*_*i*_^*s*^ be the probability that vertex *x* belongs to label *s*. The *s*-tag set is defined as *Q*(*v*_*j*_) = *s*, ∀*v*_*j*_ ∈ *U*_*s*_, where 0 < *s* ≤ *K*, and *K* is the number of all seed points. For ∀*v*_*j*_ ∈ *U*_*s*_, define

(24) $$\begin{array}{c} {m_{j}}^{s}=\left\{\begin{array}{cc} 1, & \text{when }Q\left(v_{j}\right)=s,\\ 0, & \text{when }Q\left(v_{j}\right)\neq s. \end{array}\right. \end{array}$$

The solution to the Dirichlet problem is *L*_*U*_*X* = −*B*^*T*^*M*. The sum of all probabilities in which any vertex is satisfied is 1, that is, ∑_*s*_*x*_*i*_^*s*^ = 1, ∀*v*_*i*_ ∈ *V*.

## 4. Experiments and Results

We randomly selected 12 sets of TOF-MRA data from the open head magnetic resonance data set [19] on the network to verify the reliability of the proposed method. The data were generated by an MRI scanner under 3T, the data sampling interval was was 0.5 mm × 0.5 mm × 0.8 mm, and the corresponding image size was 448 × 448 × 128. Each set of data was manually segmented by a medical imaging specialist as the gold standard for evaluation. The test environment was an Intel(R) Core(TM) i7-6700 CPU @ 3.40 GHz and 3.41 GHz CPU processor with a total memory of 16 GB. Data preprocessing and segmentation were performed in Visual Studio 2017 and MATLAB 2017.

The Dice coefficient was chosen as the empirical similarity measure. Moreover, Marching Cubes [20] was chosen to fill the extracted outline of each slice to generate a binary 3D segmentation. The Dice coefficient *D*(*A*_*R*_, *B*_*GT*_) is defined as follows:

(25) $$\begin{array}{c} D\left(A_{R},B_{GT}\right)=\frac{2\left(A_{R}\cap B_{GT}\right)}{A_{R}+B_{GT}}, \end{array}$$

where *A*_*R*_ is the result of the segmentation and *B*_*GT*_ is the gold standard. A value close to 1 indicates a good segmentation result, whereas a value close to 0 indicates a poor consensus.

Many blood vessels, especially veins, are located in the border area of the brain. A small difference in the boundary area between the two segments does not result in a strong change in the similarity measure described above. Therefore, even if the Dice coefficient implies a good consensus, these similarity measures cannot provide information about the blood vessels involved in the segmentation. Given that the pretreatment step as an improved vessel segmentation and visualization is one of the main tasks of the proposed method, the FPR and FNR parameters are introduced herein to quantify the inclusion rate of vascular voxels by the automatic segmentation of the blood vessels.

(26) $$\begin{array}{c} \left\{\begin{array}{c} \text{FDR}\left(A_{R},B_{GT}\right)=\frac{A_{R}\cap {B_{GT}}^{C}}{A_{R}+B_{GT}},\\ \text{FNR}\left(A_{R},B_{GT}\right)=\frac{{A_{R}}^{C}\cap B_{GT}}{B_{GT}}. \end{array}\right. \end{array}$$

Before processing, a set of preprocessing experiments was designed to reduce image quality and images from background areas, such as nonbrain tissue. Each TOF image sequence was first preprocessed using the histogram-based plate boundary artifact reduction method proposed by Kholmovski et al. [21] to reduce slice-related intensity variations caused by multiplate acquisition. Next, the N3 algorithm is used to correct for in-slice intensity intensities caused by poor RF coil uniformity [22].

**Figure 6:**
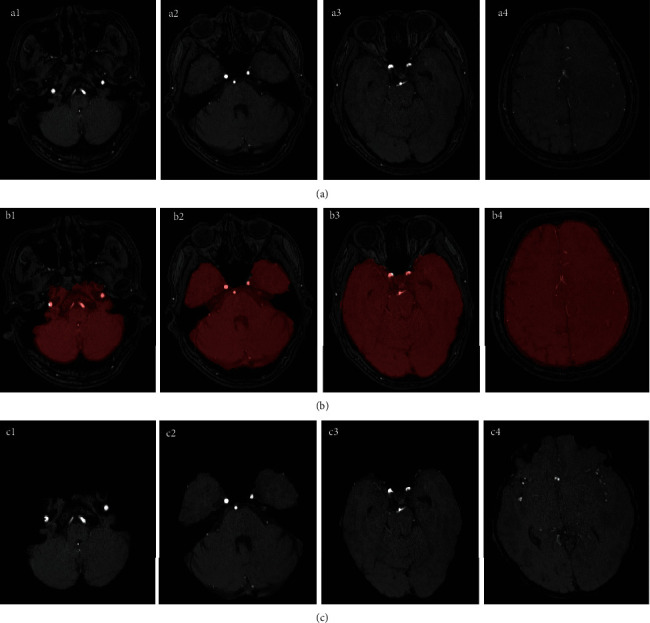
Brain extraction: (a1–a4) an example of data at different slices; (b1–b4) detected brain mask at different slices; (c1–c4) extracted brain at different slices.

Figure 6 shows the results of brain extraction at different slices. The maximum value of the mixed model Gaussian distribution was used as the threshold. Following the work of Forkert et al., the parameter settings of this paper are *ϖ*_*GM*_ = 0.2, *ϖ*_*GD*_ = 0.4, and *ϖ*_*DE*_ = 0.8, which show good performance on MRA cerebrovascular images. The mask ([0‐1]) obtained in this paper was first expanded to ensure that the blood vessels in the marginal region of the brain can be contained. The final mask was logically ANDed with the original image, as Equation (27). The expanded connected domain was 9 and the expansion coefficient was 3.

(27) $$\begin{array}{c} A⊕B=\left\{x,y,z\;|\;{\left(B\right)}_{xyz}\cap A\neq \emptyset \right\}. \end{array}$$

**Figure 7:**
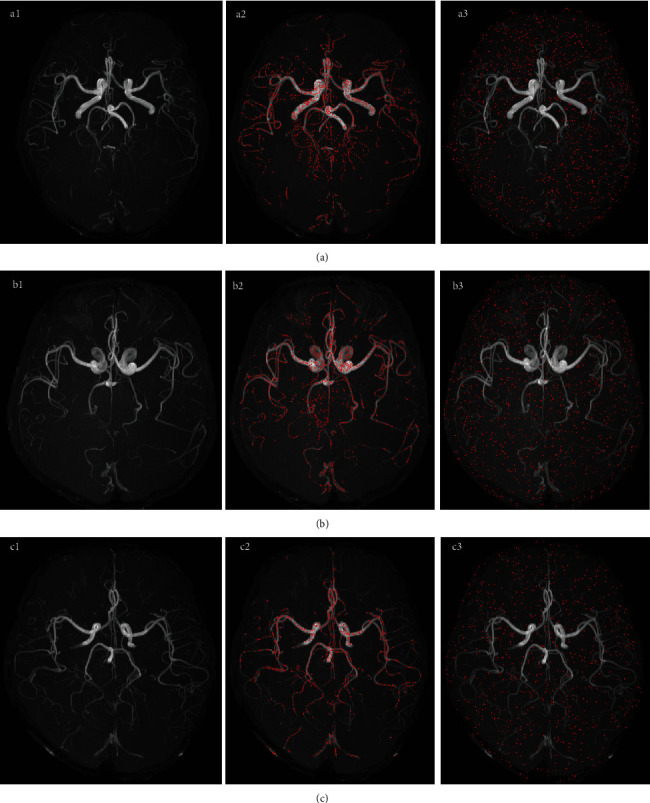
Examples of detected seed points: (a1–a3) maximum projection of original image; (b1–b3) detected vascular seed points; (c1–c3) detected background seed points.

The local maximum point of *I* > Thr in the maximum intensity projection map was selected for the target seed point; here, *I* represents the gray value of the projected image and Thr is the selected threshold. The selection of thresholds follows the principle of including as many targets as possible. The local maximum ensures that the seed point is valid and nonredundant. Avoid vascular information covering by projection, projecting three axes and removing duplicate points. The constraints of the connected domain can prevent the seed point set from including interference factors, such as noise. The entire set of seed points contains a set of blood vessel seed points and a set of background (nonvascular) seed points (Figure 7).

**Figure 8:**
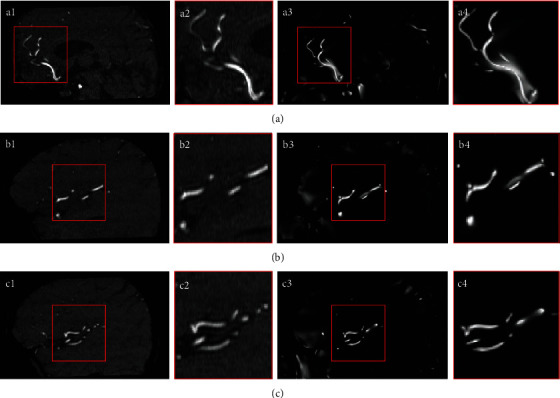
Vascular structures and their corresponding enhanced results: (a1–c1) original vascular structures; (a2–c2) enlarged vessels; (a3–c3) enhanced results of (a1–c1); (a4–c4) enlarged enhanced results.

Length-strained enhancement was applied to show the enhanced contrast of blood vessels and improve visualization.  Figure 8 shows the extracted enhanced results of the three sets of data, and details are shown by expanding the window. *L*_max_ = 100, *L*_min_ = 9, and *γ* = 3 were chosen in the present study on the basis of a previous work [17]. Here, the original image clearly has more background interference than the processed one. The contrast of the target area can be effectively improved through length-strained enhancement, and tissue interference, such as the brain, spinal cord, and fat, can be filtered. Comparison of the experimental data of Figures 8(a1)–8(a4) and Figures 8(b1)–8(b4) reveals that the method has no limitation on the blood vessels of different sizes. Therefore, if the scale threshold can be discriminated in the subsequent processing, the method can also effectively filter out arteriovenous information. Figures 8(c1)–8(c4) show that the limited length inhibition can increase the vascular recognition degree and incorrect expansion of the tissue blood vessel. However, the blood vessels in vascular enhanced images tend to be narrower than the original data set, which is attributed to several factors. At the boundary of the blood vessel, the vessel's vesselness is not strong enough. This phenomenon may cause misjudgment of a certain background area, as shown in Figures 8(c2) and 8(c4). This defect will be resolved by random walk segmentation.

**Figure 9:**
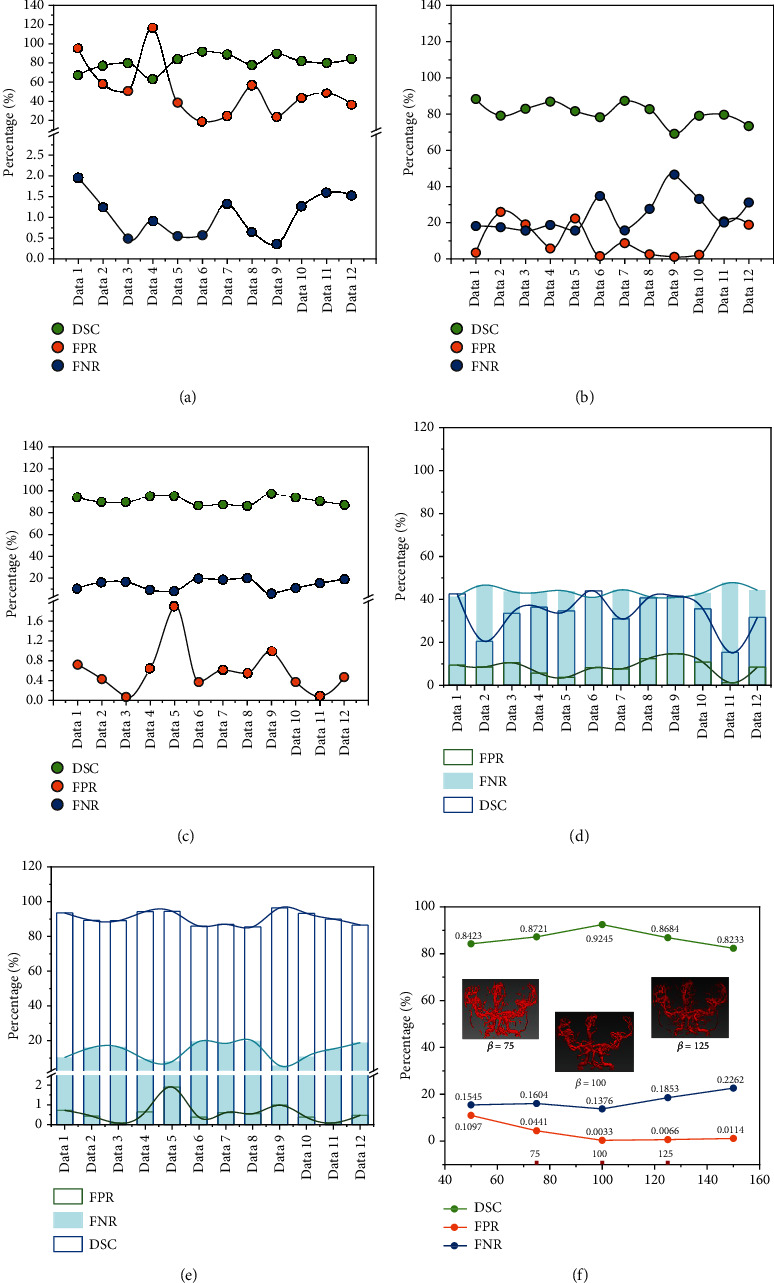
Results of three sets of comparative experiments. (a–c) The results of the Chapman algorithm, Forkert algorithm, and proposed algorithm, respectively. (d, e) The results tested without enhancement and tested after enhancement, respectively. (f) The evaluation of the *β* parameters. DSC, FPR, and FNR are given as green, yellow, and blue curves, respectively.

To evaluate the performance of the proposed method, two traditional vascular segmentation algorithms, the Chapman algorithm [23] and the Forkert algorithm [24], are introduced to compare with our method. Here, three sets of comparative experiments were set up, and the experimental results are shown in Figure 9. Among them, Figure 9(a) is the results of the Chapman algorithm, Figure 9(b) is the results of the Forkert algorithm, and Figure 9(c) is the results of the proposed algorithm. Figures 9(d) and 9(e) give the results tested without enhancement or tested after enhancement, respectively. Among them, the blue hollow histogram is DSC, the green hollow histogram is FPR, and the blue solid histogram is FNR. Finally, Figure 9(f) shows the results of an evaluation of the *β* parameters. Among these figures, DSC, FPR, and FNR are given as green, yellow, and blue curves, respectively. Tables 1–3 show the quantitative results of these three sets of comparative experiments.

Table 1 gives the comparative experimental results of the Chapman algorithm, Forkert algorithm, and our method. Here, these three algorithms need seed points in the procedure of segmentation. In the Chapman algorithm and Forkert algorithm, seed points and multifeatures of the original image are combined to extract the structures of blood vessels. In the present work, consistent seed points were controlled as a fixed variable of the three-group segmentation method to eliminate the interference of subjective factors. The parameters used in the proposed method were determined on the basis of recent research [25] and experimental verification. The parameters used in this paper are *ϖ*_*GM*_ = 0.2, *ϖ*_*GD*_ = 0.4, *ϖ*_*DE*_ = 0.8, *L*_max_ = 100, *L*_min_ = 9, *γ* = 3, and *β* = 100. Finally, the resulting data based on brain segmentation in the TOF-MRA image were obtained.

**Figure 10:**
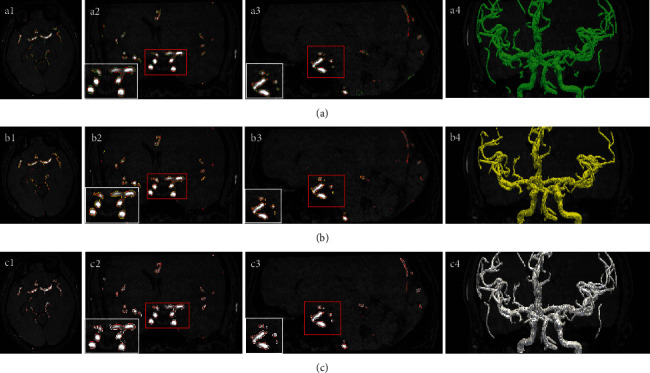
Segmentation results and their local segmentation details of the (a1–a4) Chapman algorithm, (b1–b4) Forkert algorithm, and (c1–c4) proposed method.

Table 1 lists the results of the evaluation subdivision of the proposed method. The average Dice coefficient was 90.68% compared with 80.17% and 80.70% of the other two groups of control experiments. Moreover, FPR and FNR were 0.57% and 13.30%, respectively. In terms of segmentation results (Figure 10), obtaining a small blood vessel branch by using Forkert et al.'s method is difficult because of the insufficient judgment of the details (Figures 10(b1)–10(b4)), and its FNR index is 24.56%. The method of Chapman et al. cannot effectively exclude the interference of the image (Figures 10(a1)–10(a4)), and the background area is insufficiently judged with an FPR index of 50.67%. The standard deviation of DSC is 0.037734391. The dispersion of data is stable; hence, the proposed method has good robustness [26]. Overall, the method proposed in the present work achieves good performance. By employing the random walk algorithm, this work proposes a fully automated method including automatic acquisition of seeds. Partially complex vascular regions require accurate clinical experience for judgment, and ensuring adequate target labeling by relying only on reasonable threshold selection is difficult. Therefore, the high FNR index obtained is attributed to the missing seed points. While the result is still lower than that of the control test in Forkert's group, the accuracy of the method generally meets clinical requirements. If higher accuracy is required, the clinician and related staff can manually calibrate the seed point in combination with clinical experience.

To verify the importance of length-strained enhancement, a compared nonenhancement experiment was designed as a control group, and the results are shown in Table 2. The method requires three steps: (1) skull stripping, (2) seed point selection, and (3) random walk segmentation. A single variable was controlled, and all parameters had the same value. The results of comparative verification under this premise are shown in Table 2. Effective contrast between the target and the background cannot be achieved due to the lack of length-strained enhancement. Thus, accurately finding the structure of the blood vessel by relying on the gray limit of random walk is difficult.

The last set of experiments verified the parameters of the random walk, as shown in Table 3. The random walk algorithm designed in this paper establishes weights based on gray values; thus, choosing different *β* parameter values will yield different results. As such, a comparison experiment of *β* value parameters was designed. Here, *β* had a range of 50–150 and an interval of 25. Two sets of data were selected as the verification result. The results in Table 3 indicate that the final selected parameter value is 100.

It is worth mentioning that in the process of the random walker, a large number of sparse matrices need to be calculated in the process of obtaining the segmentation probability through the *L* matrix. Hence, sufficient memory and computing are necessary to obtain the result in clinical experiments.

## 5. Conclusions

This work presents a method for automatically segmenting cerebrovascular vessels in 3D TOF-MRA images. Three sets of control experiments and a set of parameter verification experiments were designed to evaluate the proposed method. Two of the control experiments were compared with two traditional methods, and another set of control experiments was compared with their own variables. The results show that the proposed method can achieve good accuracy. The image obtained after vascular enhancement provides good results for the spectral band provided by the random walk point. The proposed method takes into account the structural properties of blood vessels in the TOF-MRA image and constructs a set of seed points with appropriate thresholds. The final result leads to a large FNR indication under the premise of satisfying the accuracy, which is caused by the insufficient selection of seed points. Actual conditions may have poor contrast, such as lesions, noise, and quality blur. If high accuracy is required, the seed point can be manually selected by the clinician and first-time staff. Combining this technique with clinical knowledge can lead to precise results.

## Figures and Tables

**Table 1:** The comparative results of the Chapman algorithm, Forkert algorithm, and proposed method, respectively.

Data	Chapman algorithm			Forkert algorithm			Proposed algorithm		
	FPR	FNR	DSC	FPR	FNR	DSC	FPR	FNR	DSC
*V*_1_	94.69%	1.95%	66.99%	3.45%	18.17%	88.33%	0.33%	13.76%	92.45%
*V*_2_	58.05%	1.25%	76.90%	25.97%	17.53%	79.13%	0.43%	16.02%	89.24%
*V*_3_	50.72%	0.49%	79.52%	19.07%	15.63%	82.94%	0.12%	11.75%	92.80%
*V*_4_	115.85%	0.92%	62.92%	5.77%	18.73%	86.90%	0.64%	9.21%	94.34%
*V*_5_	38.57%	0.55%	83.56%	22.32%	15.64%	81.63%	1.90%	8.03%	94.48%
*V*_6_	18.53%	0.57%	91.24%	1.53%	34.72%	78.27%	0.37%	19.55%	85.99%
*V*_7_	24.44%	1.32%	88.46%	8.72%	15.75%	87.31%	0.61%	18.45%	86.94%
*V*_8_	56.75%	0.65%	77.58%	2.55%	27.64%	82.74%	0.55%	19.89%	85.55%
*V*_9_	23.46%	0.36%	89.32%	1.13%	46.58%	69.13%	0.98%	5.70%	96.39%
*V*_10_	42.94%	1.26%	81.71%	2.31%	33.14%	79.05%	0.37%	10.86%	93.33%
*V*_11_	48.23%	1.59%	79.80%	20.75%	20.08%	79.65%	0.09%	15.31%	90.02%
*V*_12_	35.81%	1.52%	84.06%	18.85%	31.15%	73.36%	0.47%	18.89%	86.59%
Mean	50.67%	1.04%	80.17%	11.04%	24.56%	80.70%	0.57%	13.95%	90.68%

**Table 2:** The comparative results tested with nonenhancement and tested after enhancement (proposed algorithm).

Data	Nonenhancement			Proposed algorithm		
	FPR	FNR	DSC	FPR	FNR	DSC
*V*_1_	9.38%	41.23%	42.58%	0.33%	13.76%	92.45%
*V*_2_	8.54%	46.68%	20.43%	0.43%	16.02%	89.24%
*V*_3_	10.52%	43.67%	33.50%	0.12%	11.75%	92.80%
*V*_4_	5.73%	43.32%	36.36%	0.64%	9.02%	94.34%
*V*_5_	3.72%	43.89%	34.67%	1.90%	8.03%	94.48%
*V*_6_	8.21%	40.93%	43.98%	0.37%	19.55%	85.99%
*V*_7_	7.66%	44.49%	30.99%	0.61%	18.45%	86.94%
*V*_8_	1.24%	41.45%	40.74%	0.55%	19.89%	85.55%
*V*_9_	1.47%	40.94%	41.49%	0.98%	5.70%	96.39%
*V*_10_	10.76%	43.08%	35.66%	0.37%	10.86%	93.33%
*V*_11_	1.23%	4.78%	15.36%	0.08%	15.31%	90.02%
*V*_12_	8.42%	44.28%	31.65%	0.47%	18.89%	86.59%
Mean	6.41%	39.90%	33.95%	0.57%	13.94%	90.68%

**Table 3:** Two sets of verification data with *β* having a range of 50-150.

Beta	Data 1			Data 2		
	DSC	FPR	FNR	DSC	FPR	FNR
50	84.23%	10.97%	15.45%	90.66%	5.15%	11.17%
75	87.21%	4.41%	16.04%	91.72%	1.23%	1.25%
100	92.45%	0.33%	13.76%	92.84%	0.12%	11.75%
125	86.84%	0.66%	18.53%	73.62%	0.0689%	29.43%
150	82.33%	1.14%	22.62%	53.25%	40.48%	28.08%

## Data Availability

The source data included in this paper was downloaded from the open head magnetic resonance data set on the Internet, and it is available for everyone. The link of the data can be found in Ref. [19].
